# Erste klinische und onkologische Erfahrungen mit der Triplet-Therapie beim „high-volume“ metastasierten hormonsensiblen Prostatakarzinom

**DOI:** 10.1007/s00120-023-02253-8

**Published:** 2023-12-21

**Authors:** Mike Wenzel, Benedikt Hoeh, Jan Kasparek, Clara Humke, Sophie von Koskull, Felix K. H. Chun, Séverine Banek, Philipp Mandel

**Affiliations:** Klinik für Urologie, Universitätsklinikum Frankfurt, Theodor-Stern-Kai 7, 60590 Frankfurt, Deutschland

**Keywords:** mHSPC, PEACE 1, ARASENS, Abirateron, Darolutamid, mCRPC, mHSPC, PEACE 1, ARASENS, Abiraterone, Darolutamide, mCRPC

## Abstract

**Hintergrund:**

Die Behandlung mittels Androgendeprivationstherapie (ADT) plus erweiterter Hormontherapie (ARTA) stellt die Standardtherapie beim metastasierten hormonsensiblen Prostatakarzinom (mHSPC) dar. Neue Daten von Triplet-Kombinationstherapien aus ADT + ARTA (Abirateron/Darolutamid) + Docetaxel-Chemotherapie zeigten einen Überlebensvorteil für gewisse mHSPC-Patientengruppen.

**Fragestellung:**

Welches Therapieansprechen ist im Real-world-mHSPC-Setting mittels Triplet-Kombinationstherapie zu erwarten und welche Nebenwirkungen treten gehäuft auf?

**Ergebnisse:**

Alle Patienten, die eine Triplet-Kombinationstherapie aus ADT + ARTA (Abirateron/Darolutamid) + Docetaxel erhalten haben, wurden für die vorliegende Studie eingeschlossen. Insgesamt konnten 14 Patienten mit einem medianen Alter von 62 Jahren und 10/14 Abirateron- bzw. 4/14 Darolutamid-Therapien inkludiert werden. Der mediane PSA vor Therapiebeginn lag bei 77 (IQR 44–150) ng/ml. Insgesamt hatten 86 % der Patienten einen PSA-Abfall > 90 % unter Therapie und der mediane PSA-Nadir lag bei 0,3 ng/ml. Schwerwiegende Nebenwirkungen (Grad III) unter der Triplet-Therapie traten bei insgesamt 2 Patienten (14,2 %) auf mit fieberhafter Neutropenie 7,1 % (1/14) bzw. Gastroenteritis und Infektgeschehen 7,1 % (1/14). Leichtgradige Nebenwirkungen (Grad I/II) wie Polyneuropathie (1/14), Mukositis (1/14), Xerostomie (1/14), Gewichtsverlust (1/14) und Fatigue (3/14) wurden ebenso detektiert. Die Chemotherapie wurde bei einem Patienten aufgrund von Nebenwirkungen unterbrochen. Nach einem medianen Follow-up von 10 (IQR: 7–17) Monaten zeigten sich 2 Patienten (14,2 %) mit Progression zu einer Kastrationsresistenz.

**Zusammenfassung:**

Die Triplet-Therapie zeigt sich im klinischen Alltag mit einem sehr guten PSA-Ansprechen. Nebenwirkungen unter der Therapie sind v. a. durch die klassische Chemotherapie getriggert.

## Abstract (en)

**Background:**

Treatment with androgen deprivation therapy (ADT) plus extended hormone therapy (ARTA) is the standard of care for metastatic hormone-sensitive prostate cancer (mHSPC). Recent data of triplet combination therapies of ADT + ARTA (abiraterone/darolutamide) + docetaxel chemotherapy showed a survival advantage for specific mHSPC patient subgroups.

**Purpose:**

What treatment response is observed in real-world mHSPC setting using triplet combination therapy and what are the expected side effects?

**Results:**

All patients receiving triplet combination therapy of ADT + ARTA (abiraterone/darolutamide) + docetaxel were included in the current study. A total of 14 patients with a median age of 62 years and 10/14 abiraterone or 4/14 darolutamide therapy could be included. The median PSA before initiation of therapy was 77 ng/ml (IQR 44–150). Overall, 86% of patients had a PSA response > 90% and the median PSA nadir was 0.3 ng/ml. Severe adverse events (grade III) during triplet therapy occurred in two patients (35,7%) with respectively febrile neutropenia 7.1% (1/14) and diarrhea with infection 7.1%. Other low grade adverse events (grade I/II) consisted of polyneuropathy (1/14), mucositis (1/14), xerostomia (1/14), weight loss (1/14) and fatigue (3/14) were detected. Chemotherapy was interrupted in one patient due to adverse events. After a median follow-up of ten months (IQR: 7–17), two patients (14.2%) showed progression to castration resistance.

**Conclusion:**

Triplet therapy shows a very good PSA response in clinical practice. Adverse events during therapy are mainly triggered by classical chemotherapy-known side effects.

## Einleitung

Die kombinierte Androgendeprivationstherapie (ADT) in Kombination mit einem neuen Androgenrezeptorantagonisten (ARTA) stellt aktuell den Behandlungsstandard für das metastasierte hormonsensitive Prostatakarzinom (mHSPC) dar [1–5]. Aktuell werden im klinischen urologischen Alltag vornehmlich Metastasierungsmuster nach der CHAARTED-Studie (Low- vs. High-volume-Metastasierung) und der LATITUDE-Studie (Low- vs. High-risk-Erkrankung) zwecks Risikoklassifikation des mHSPC genutzt [6–8]. High-volume-Metastasierung ist hierbei definiert als ≥ 4 ossäre Metastasen, wobei mindestens eine außerhalb des knöchernen Achselskeletts befindlich sein muss oder mindestens einer viszeralen Metastase. Als High-risk-mHSPC sind mindestens zwei der folgenden drei Kriterien zu erfüllen: ≥ 3 ossäre Metastasen, Gleason Score 8–10 und mindestens eine viszerale Metastase.

Die initiale Docetaxel-Chemotherapie im mHSPC hat in der damaligen CHAARTED-Studie einen Gesamtüberlebensvorteil in der High-volume-mHSPC-Kohorte sowie im STAMPEDE-Arm C für die Low- und High-volume-mHSPC-Kohorte gezeigt, sodass diese Therapie hier angewendet wird [6, 9]. Abirateron zeigte zwar eine Wirksamkeit bezüglich des Gesamtüberlebens bei allen Patienten (LATITUDE: nur High-risk-mHSPC eingeschlossen und STAMPEDE-Arm G: Vorteil beim Low- und High-volume-mHSPC), ist allerdings nur für das High-risk-mHSPC zugelassen [7, 10]. Dem gegenüber abzugrenzen sind die „ARTA-Allcomer“ Enzalutamid und Apalutamid, welche unabhängig der oben genannten Metastasierungsmuster zugelassen sind [11–13].

Im weiteren Verlauf wurden 2021 bzw. 2022 erstmals Daten der PEACE-1- und ARASENS-Studie bzgl. einer Triplet-Therapie durch die Kombination aus ADT, ARTA und Docetaxel-Chemotherapie im Vergleich zur ADT und Docetaxel Chemotherapie veröffentlicht. Hierbei zeigte sich im PEACE-1-Trial (ADT + Abirateron + Docetaxel) und bei der ARASENS-Studie (ADT + Darolutamid + Docetaxel) ein Überlebensvorteil, sodass entsprechend der ARASENS-Daten (jedoch nicht für die PEACE-1-Daten, hier erfolgt die Anwendung entsprechend einer Off-label-Therapie) eine Zulassung für alle Patienten im mHSPC erfolgte [14, 15].

Besonders interessant neben dem Endpunkt des Überlebensvorteils in den Triplet-Studien war die Toxizität im Vergleich zur alleinigen Kombination aus ADT + Docetaxel-Chemotherapie. Beispielsweise zeigte sich in der ARASENS-Studie sowohl in der Vergleichs- als auch Verumgruppe eine 4 %ige Grad-5-Toxizität; entsprechend einer Sterbewahrscheinlichkeit jedes 25. Patienten unter Therapie. Bekanntermaßen zeigen sich immer wieder Diskrepanzen zwischen Daten aus prospektiven randomisierten Studien und der im Alltag beobachteten Real-world-Evidenz, welche oftmals aufgrund einer Verzerrung der Patientenselektion herrührt.

Die folgende Arbeit beschäftigt sich somit mit der Frage, in welcher Reihenfolge die Triplet-Therapie verabreicht werden kann, welche kurzfristigen onkologischen Ansprechraten und welches Nebenwirkungsprofil nach Einführung der Triplet-Therapien gemäß des PEACE-1- und ARASENS-Schemata im klinischen urologischen Alltag zu sehen und erwarten sind.

## Material und Methoden

### Patientenkohorte

Für die aktuelle Studie wurden nach vorherigem Ethikvotum alle mHSPC-Patienten mit Triplet-Therapie bestehend aus ADT, ARTA (Darolutamid oder Abirateron) sowie Docetaxel-Chemotherapie retrospektiv aus der metastasierten Prostatakarzinomdatenbank der Klinik für Urologie der Goethe-Universitätsklinikum Frankfurt, Deutschland, eingeschlossen. Einschlusszeitraum der Studie war von 11/2021 bis 04/2023. Alle Patienten‑, Tumorcharakteristika sowie onkologischen Informationen wurden aus den Patientenakten anonymisiert entnommen. Patienten mit einem Follow-up < 3 Monaten wurden von der Studienauswertung ausgeschlossen.

### Triplet-Therapie

Patienten mit Triplet-Therapie für das mHSPC erhielten eine Androgendeprivation mittels LHRH(„luteinizing-hormone-releasing hormone“)-Antagonist oder -Agonisten. Kombiniert wurde dies mit Abirateron (1000 mg, einmal täglich sowie 5 mg Prednisolon) oder Darolutamid 600 mg (300 mg 2‑mal täglich) als Dauertherapie bis zum Therapieversagen. Zudem erfolgte eine begleitende intravenöse Chemotherapie mit Docetaxel 75 mg/kg^2^ im 3‑wöchigen Intervall mit bis zu 6 Zyklen. Die Entscheidung zwischen dem Einsatz von Darolutamid oder Abirateron erfolgte nach der Darolutamid-Zulassung 03/23 nach entsprechender Aufklärung des Patienten über mögliche Vor- und Nachteile.

Nach Initiierung der ADT wurde parallel die ARTA-Therapie eingeleitet. Die erstmalige Chemotherapie erfolgte 2–3 Wochen nach ARTA-Initiierung.

### Statistische Auswertung und Studienendpunkte

Die deskriptive Datenauswertung enthielt Häufigkeitsverteilungen für kategorische Variablen sowie Medianberechnungen inklusive Interquartilsabstände (IQR) sowie Gesamtverteilung (Range) für kontinuierliche Variablen. Alle Auswertungen erfolgten mit der Statistiksoftware R (Version 4.1.2., R Foundation for Statistical Computing, Wien, Österreich).

Als kurzfristiges onkologisches Therapieansprechen unter Triplet-Therapie wurde der absolute PSA-Nadir sowie der relative PSA-Nadir in Abhängigkeit zum PSA-Wert bei vor Therapiebeginn herangezogen. Zwecks des Nebenwirkungsprofils der Triplet-Therapie wurden Therapieunterbrechungen, als auch die Häufigkeiten und Schweregrade der Nebenwirkungen evaluiert. Als Therapieversagen unter Triplet-Therapie wurde der bildmorphologische oder laborchemische Übergang in ein metastasiertes kastrationsresistentes Prostatakarzinom definiert [16].

## Ergebnisse

Insgesamt konnten 14 mHSPC-Patienten im Zeitraum von 11/2021 bis 04/2023 für die vorliegende Studie eingeschlossen werden, die eine Triplet-Therapie gemäß der PEACE-1- oder ARASENS-Studie erhielten. Alle Patienten hatten hierbei eine High-volume-Metastasierung gemäß CHAARTED-Studie und davon hatten 12 (86 %) Patienten ein High-risk-mHSPC gemäß LATITUDE-Studie vorliegend. Insgesamt erhielten 10 Patienten eine Therapie aus ADT + Abirateron + Docetaxel (71 %) und 4 Patienten ADT + Darolutamid + Docetaxel (29 %). Als ADT erhielten 12 Patienten Leuprorelin (86 %) und 2 mHSPC-Patienten Triptorelin (14 %).

### Patienten- und Tumorcharakteristika

Das mediane Alter der eingeschlossenen Patienten betrug 62 (IQR: 55–69, Range: 44–79) Jahre mit einem ECOG von 0–1 in 92,9 % der Fälle (Tab. 1). Bezüglich des Tumorgradings zeigten 13 Patienten (92,9 %) ein Gleason Score 8–10 in den diagnostischen histopathologischen Präparaten. Eine initiale Lokaltherapie mittels radikaler Prostatektomie wurde bei einem Patienten durchgeführt (pT3a, pN0, R1, Gleason Score 9), bei welchem sich im Verlauf eine sekundäre Metastasierung (7,1 %) entwickelte. Alle anderen Patienten wiesen ein primäres mHSPC auf (92,9 %).

**Tab. 1:** Charakteristika von Patienten mit Tripple-Therapie beim „high-volume“ metastasierten hormonsensiblen Prostatakarzinom (mHSPC; stratifiziert nach Therapieregime)

Variable		Gesamtkohorte (*n* = 14)	Darolutamid + Docetaxel (*n* = 4)	Abirateron + Docetaxel (*n* = 10)
Alter ED PCa (Jahre)	Median (IQR)	62 (54–69)	63 (56–69)	62 (54–68)
PSA mHSPC (ng/ml)	Median (IQR)	77 (44–150)	120 (61–2375)	71 (44–98)
PSA-Abfall nach 1. Zyklus Docetaxel (%)	Median (IQR)	−90 (−73–(−97))	−96 (−80–(−97))	−90 (−76–(−97))
PSA-Nadir mHSPC (ng/ml)	Median (IQR)	0,3 (0,1–2,3)	6,3 (0,3–12,5)	0,2 (0,1–0,5)
Follow-up (Monate)	Median (IQR)	10 (7–17)	5 (5–6)	15 (10–18)
ECOG-Status (%)	0	9 (64,3)	1 (25)	8 (80)
	1	4 (28,6)	3 (75)	1 (10)
	≥ 2	1 (7,1)	0 (0)	1 (10)
Staging (%)	Konventionell	11 (78,6)	3 (75)	8 (80)
	PSMA-PET/CT	3 (21,4)	1 (25)	2 (20)
PSA-Abfall > 90 % unter Triplet-Therapie	–	13 (85,7)	3 (75)	9 (90)

*ED* Erstdiagnose, *PCa* Prostatakarzinom, *PSA* prostataspezifisches Antigen, *ECOG* Eastern Cooperative Oncology Group, *mHSPC* metastasiertes hormonsensibles Prostatakarzinom, *PSMA* prostataspezifisches Membranantigen, *PET* Positronenemissionstomographie

Im initialen Staging erhielten 11 (78,6 %) der mHSPC-Patienten ein konventionelles Staging bestehend aus Computertomographie (CT) und Skelettszintigraphie, während 3 Patienten ein PSMA-PET/CT (21,4 %) bekamen. Insgesamt zeigten 7 mHSPC-Patienten > 10 ossäre (50 %) und 2 Patienten mindestens eine viszerale Metastase (14,2 %), von denen beide ein initiales konventionelles Staging erhielten (Tab. 1).

### PSA-Response

**Abb. 1:**
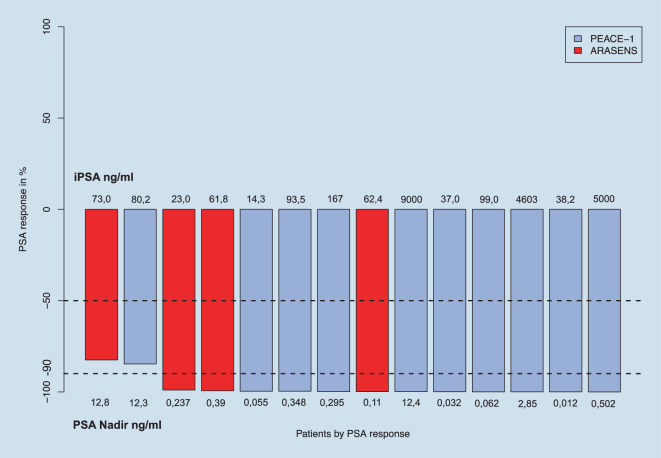
„Waterfall plot“ zur Demonstration des PSA-Ansprechens (prostataspezifisches Antigen) auf die Tripple-Therapie gemäß PEACE‑1 (ADT + Docetaxel + Abirateron) oder ARASENS (ADT + Docetaxel + Darolutamid) -Studie

Der mediane PSA bei Therapiebeginn des mHSPC lag bei 77 (IQR: 44–150, Range: 14–9000) ng/ml. Der PSA-Nadir unter Triplet-Therapie lag im Median bei 0,32 (IQR: 0,1–2,3, Range: 0,012–12,8) ng/ml nach einem medianen Follow-up von 10 Monaten. Ein relativen PSA-Abfall von > 90 % respektive 99 % zeigten 12 (85,7 %) bzw. 11 mHSPC-Patienten (78,6 %, Abb. 1). Nach dem ersten Zyklus Chemotherapie kombiniert mit ADT + ARTA zeigte sich bereits im Median ein PSA-Abfall von −89,8 % (IQR: −73,3 bis −97,3 %, Range: −32,9 bis −99,2 %). Zwei mHSPC-Patienten (14,2 %) entwickelten eine bildmorphologische bzw. Kastrationsresistenz im Laufe des medianen Follow-up von 10 Monaten nach Beginn der Triplet-Therapie (IQR: 7–17, Range: 4–21 Monate) mit einem PSA-Wiederanstieg auf 15,9 bzw. 30,8 ng/ml.

### Nebenwirkungsprofil und Management

Insgesamt erhielten 6 mHSPC-Patienten (42,9 %) begleitend zur Triplet-Therapie eine Radiatio mindestens einer Metastase aufgrund von ossären Schmerzexazerbationen (*n* = 5/6) oder drohender Spinalkanalkompression mit Querschnittsgefahr (*n* = 1/6, Tab. 2). Diese Therapie erfolgte jeweils innerhalb der ersten beiden Monate nach mHSPC-Diagnosestellung.

**Tab. 2:** Nebenwirkungen unter Tripple-Therapie beim „high-volume“ metastasierten hormonsensiblen Prostatakarzinom (mHSPC) sowie Salvage-Treatment (definiert als Radiatio oder Operation einer Metastase aufgrund von ossärer Schmerzexazerbationen oder drohender Querschnittgefahr bei Spinalkanalkompression)

	Gesamtkohorte (*n*)	Darolutamid + Docetaxel (*n*)	Abirateron + Docetaxel (*n*)
Patienten mit Nebenwirkungen	8	1	7
*Art der Nebenwirkung*			
Fatigue Grad I/II	3	1	2
Chemotherapie Extravasation	1	0	1
Mukositis Grad I	1	0	1
Gewichtsverlust Grad II	1	0	1
Infektionsgrad II	1	0	1
Neutropenes Fieber Grad III	1	0	1
Polyneuropathie Grad I	1	0	1
Diarrhö Grad III	1	0	1
Xerostomie Grad I	1	0 (0)	1
„Salvage Treatment“	6 (42,9)	1 (25,0)	5 (50,0)

Im Verlauf der Chemotherapie kam es bei insgesamt 8 mHSPC-Patienten zu relevanten unerwünschten Nebenwirkungen (Tab. 2). Hiervon waren 2 Patienten von höhergradigen Nebenwirkungen mit jeweils einem febrilem neutropenem Fieber (Grad III) sowie einem gastrointestinalen Infekt mit reduziertem Allgemeinzustand (Grad III) betroffen. Weitere geringgradiger Nebenwirkungen wurden bei jeweils einem Patient (je 7,1 %) mit oraler Mukositis (Nebenwirkung Grad I), leicht- bis mittelgradige Polyneuropathie der Zehen und Finger (Grad II) sowie Xerostomie (Grad I) und Gewichtsverlust (Grad II) detektiert. Fatigue-Symptomatik (Grad I–II) wurde von 3 Patienten berichtet. Zu einer Extravasation der Chemotherapie kam es in einem Fall ohne längerfristige Hauttoxizitäten (Grad I–II). Die Chemotherapie wurde bei einem mHSPC-Patienten aufgrund des gastrointestinalen Infektes mit Reduzierung des Allgemeinzustands pausiert bzw. verschoben. Nach Abschluss der Chemotherapie kam es zu keinen weiteren relevanten unerwünschten Nebenwirkungen im Verlauf unter ADT und ARTA-Therapie.

## Diskussion

Die vorliegende Studie beschäftigte sich mit dem kurzfristigen onkologischen Ansprechen von „high volume“ mHSPC-Patienten unter Triplet-Therapie bestehend aus ADT + ARTA sowie Docetaxel-Chemotherapie sowie die im Alltag auftretenden erwartbaren Nebenwirkungsereignisse. Für diese Studie wurden insgesamt 14 High-volume-mHSPC-Patienten gemäß PEACE‑1 oder ARASENS-Schemata behandelt und wichtige Erkenntnisse gewonnen.

Interessanterweise zeigte sich in der vorliegenden Studie ein klinisch bedeutsamer Unterschied hinsichtlich der allgemeinen Patienten und Tumorcharakteristika in der Real-world-Evidenz im Vergleich zu den randomisierten Phase-III-Studienkollektiven. So wiesen unsere Patienten mit einem medianen Alter von 62 Jahren ein deutlich jüngeres Alter (medianes Alter PEACE-1-Triplet-Kohorte: 66 Jahre, medianes Alter High-volume-Triplet-Kohorte ARASENS: 67 Jahre) bei deutlich höherem medianen PSA von 77 ng/ml vs. 14 (PEACE-1) bzw. 39 ng/ml (ARASENS) sowie einer ebenso höheren PSA-Range (hier bis zu 9000 ng/ml). Ebenso zeigten sich Unterschiede hinsichtlich der von uns beobachteten mHSPC-Patientenkohorte bezüglich ECOG-Status (ECOG 2-mHSPC-Patienten nicht eingeschlossen im ARASENS-Trial) und dem Metastasierungszeit (nur De-novo-mHSPC-Patienten im PEACE-1-Trial; [14, 15, 17]). Diese Diskrepanzen hinsichtlich der Patienten- und Tumorcharakteristika sind wichtig, da diese die Unterschiede des klinischen Alltags und der entsprechenden Studienkohorten widerspiegeln und Unterschiede in den Ergebnissen erklären können. Es ist anzunehmen, dass sich vornehmlich mHSPC-Patienten im jungen Lebensalter mit einem gutem Allgemeinzustand und hoher Tumorlast sowie und PSA-Werten für eine Triplet-Therapie entscheiden, da hierbei das längste Langzeitüberleben möglicherweise zu erwarten ist und somit therapieassoziierte Nebenwirkungen der zusätzlichen Chemotherapie des eher günstigen Nebenwirkungsprofils einer alleinigen ADT + ARTA-Therapie aufwiegen. Hinsichtlich der optimalen Patientenselektion für eine Triplet-Therapie bleibt zu sagen, dass die Ergebnisse der ARASENS-Studie nach Stratifizierung gemäß CHAARTED/LATITUDE zeigen, dass sich die Vorteile zugunsten der Triplet-Therapie im progressionsfreien (Vorteil im Low- und High-volume- als auch Low- und High-risk-Stadium) als auch Gesamtüberleben (Vorteil im High-volume- sowie Low- und High-risk-Stadium) auf bestimmte mHSPC-Subgruppen übertragen lassen. Eine entsprechende Zulassung für die Triplet-Therapie unabhängig von der Metastasenlast ist folglich für die Kombination aus Darolutamid/Docetaxel erfolgt. Allerdings zeigt sich in der ARASENS- wie auch der PEACE-1-Studie (hier kein Vorteil bzgl. Gesamtüberleben beim Low-volume-mHSPC) eine wahrscheinlich zu geringe Stichprobe der Low-volume-mHSPC-Patienten, als auch ein zu kurzes Follow-up um den Einfluss auf diese Studienpopulationen abschließend bewerten zu können. Zudem zeigte eine Post-hoc-Analyse auf dem diesjährigen ASCO-GU 2023, dass womöglich der Effekt der Triplet-Therapie aus Abirateron/Docetaxel bei Patienten ≥ 70 Jahren geringer ausgeprägt sein könnte und somit bei diesen Patienten ein ausgiebiges prätherapeutisches geriatrisches Assessment empfohlen werden sollte.

Zweitens zeigte sich in unserer Studie hinsichtlich des onkologischen Ansprechens ein PSA-Nadir der Patienten bei im Median 0,3 ng/ml mit einem relativen PSA-Abfall von 90 % bzw. > 99 % bei 86 und 79 % der High-volume-mHSPC-Patienten. Diese Daten lassen sich leider nicht mit den mittelfristigen onkologischen Überlebensanalysen (progressionsfreies Überleben bzw. Gesamtüberleben) der PEACE-1- oder ARASENS-Studie vergleichen, lassen allerdings vermuten, dass bei einem medianen Follow-up von 10 Monaten ein adäquates Therapieansprechen besteht. Vergleichend mit anderer Literatur der PSA-Kinetik im Setting des mHSPC zeigte z. B. eine Post-hoc-Analyse der LATITUDE-Studie (Arm ADT + Abirateron) eine 79 % Ansprechrate mit einem relativen PSA-Abfalls von mindestens 90 % und einem medianen PSA-Nadir von 0,09 ng/ml nach 6 Monaten [18]. Weiterhin wird in den EAU-Leitlinien und der Literatur auf einem PSA-Nadir von ≤ 0,2 ng/ml hingewiesen, der beim mHSPC mit einem verbessertem Gesamtüberleben assoziiert sein soll [1, 19, 20]. Andere retrospektive Studien zogen hierfür einen noch tieferen PSA-Nadir von ≤ 0,05 ng/ml heran [21]. Weiterhin zeigte sich in unserer Studie bereits ein medianer PSA-Abfall von 90 % nach dem ersten Zyklus Chemotherapie in Kombination mit ADT und ARTA, wohingegen wieder andere retrospektive Studien zeigten, dass das Erreichen des PSA-Nadirs im mHSPC mit einer dualen Therapie aus ADT + ARTA zwischen 3–5 Monaten verlängert liegt [22, 23]. Insgesamt sind die Vergleiche der PSA-Kinetik zwischen unseren Daten und den beschriebenen Studiendaten schwierig, da die zitierten Studien sich lediglich auf duale Kombinationstherapie mit ADT + ARTA oder ADT + Chemotherapie stützen und ein heterogenes Patientenkollektiv inkludierten im Vergleich zur vorliegenden Studie mit ausschließlich High-volume-mHSPC-Patienten, welche möglicherweise aufgrund der Metastasenlast und absoluten PSA-Höhe andere PSA-Kinetiken aufweisen könnten.

Im Vergleich des Nebenwirkungsprofils zeigten sich in unserer High-volume-mHSPC-Kohorte bei insgesamt 8 von 14 Patienten nennenswerte Nebenwirkungen, wovon 2 Patienten Grad-III-Nebenwirkungen entwickelten. Die Ausprägung dieser Nebenwirkungen war interindividuell stark unterschiedlich. Insgesamt zeigen die berichteten Nebenwirkungen allerdings einen starken Zusammenhang mit der begleitenden Docetaxel-Chemotherapie. Ein Vergleich der relativen Nebenwirkungsraten zu den Raten der Phase-III-PEACE-1- und ARASENS-Studie verbietet sich hier aufgrund unseres kleinen Patientenkollektivs. Allerdings bleibt zu berichten, dass wir keine Grad-4- und Grad-5-Nebenwirkungen beobachteten. Die von uns beschriebenen Events eines gastrointestinalen Infekts, einer Polyneuropathie, Fatigue sowie eines neutropenen Fiebers werden in der PEACE-1-(Triplet-Therapiearm) bzw. ARASENS-(Triplet-Therapiearm)Studie mit folgenden Anteilen berichtet: nicht berichtet vs. 9,5 %; 1 % vs. 11,7 %; 3 % vs. 13 %, 5 % vs. 7,8 %.

All diese berichteten Nebenwirkungen lassen sich wohl am ehesten auf die kombinierte Chemotherapie zurückführen. Eine Kombination der Triplet-Therapie im Vergleich zur Therapie aus ADT + Chemotherapie zeigt in beiden genannten PEACE-1- und ARASENS-Studien kein massiv erhöhtes Nebenwirkungsrisiko [14, 15, 17]. Die Nebenwirkungen unter einer ARTA-Therapien beim mHSPC sind nur in seltenen Fällen sehr ausgeprägt und beziehen sich am häufigsten auf das Herz-Kreislauf-System mit beispielsweise Hypertension, Flush-Symptomatik oder Elektrolytstörrungen [7, 12, 13, 24]. Insgesamt bleibt anzumerken, dass Darolutamid aufgrund seiner geringeren Penetration der Blut-Hirn-Schranke wohl grundsätzlich ein besseres Nebenwirkungsprofil zentralvenöser Events zu haben scheint [24].

Die vorliegende Studie hat einige Limitationen und erhebt nicht den Anspruch einer grundsätzlichen Evaluation des onkologischen Ansprechens der Triplet-Therapie beim High-volume-mHSPC. Ebenso bleibt unklar, ob der relative hohe Anteil an Patienten mit begleitender Radiatio/Metastasenchirurgie aufgrund von Schmerzexazerbationen repräsentativ für Patienten unter Triplet-Therapie ist. Angesichts der kleinen Patientenkohorte als auch des relativ kurzen Follow-up soll die Studie vielmehr erste klinischen Daten zur Anwendung präsentierten. Ebenso haben einige hier inkludierte Patienten ein PSMA-PET/CT zwecks initialer Staginguntersuchung erhalten und wurden als High-volume-mHSPC klassifiziert. Der Vollständigkeit halber bleibt zu erwähnen, dass alle Stadieneinteilungen in der CHAARTED- und LATUTIDE-Studie mittels konventioneller Bildgebungen durchgeführt wurden.

## Fazit und Praxisrelevantes

High-volume-mHSPC-Patienten die sich einer Triplet-Therapie aus ADT + ARTA (Abirateron/Darolutamid) + Docetaxel unterziehen sind im klinischen Alltag meist junge und fitte Männer mit hoher Tumorlast. Die PSA-Kinetik als Proxy eines kurzfristigen Therapieansprechens zeigt einen relativen PSA-Abfall von ≥ 99 % in > 70 % aller High-volume-mHSPC-Patienten. Nebenwirkungen unter der Triplet-Therapie sind v. a. mit der begleitenden Chemotherapie assoziiert und bedürfen eines entsprechenden Monitorings während dieses Zeitraums.
